# Cytokine-associated ROS and Ca^2+^ dysregulation in severe inflammatory cardiomyopathy

**DOI:** 10.1093/cvr/cvag099

**Published:** 2026-08-30

**Authors:** Phillip Suwalski, Ainoosh Golpour, January Weiner, Nicolas Musigk, Felix Balzer, Niklas Giesa, Ali Amr, Joanna Zyla, Peter Ganz, Hafiza Nosheen Saleem, Johannes Trebing, Farbod Sedaghat-Hamedani, Benjamin Meder, Stefan M Gold, Antje Ebert, Dieter Beule, Ulf Landmesser, Bettina Heidecker

**Affiliations:** Deutsches Herzzentrum der Charité, Universitätsmedizin Berlin, Hindenburgdamm 30, 12203 Berlin, Germany; Deutsches Herzzentrum der Charité, Universitätsmedizin Berlin, Hindenburgdamm 30, 12203 Berlin, Germany; Core Unit for Bioinformatics (CUBI), Berlin Institute of Health (BIH) at Charité, Berlin, Germany; Deutsches Herzzentrum der Charité, Universitätsmedizin Berlin, Hindenburgdamm 30, 12203 Berlin, Germany; Berlin Institute of Health (BIH) at Charité—Universitätsmedizin Berlin, Anna-Louisa-Karsch-Straße 2, 10178 Berlin, Germany; Berlin Institute of Health (BIH) at Charité—Universitätsmedizin Berlin, Anna-Louisa-Karsch-Straße 2, 10178 Berlin, Germany; TORCH - Translational Registry of Cardiomyopathies, DZHK (German Centre for Cardiovascular Research), Berlin, Germany; Department of Data Science and Engineering, Silesian University of Technology, Gliwice, Poland; Department of Medicine, University of California San Francisco (UCSF), San Francisco, USA; Department of Cardiology and Pneumology, Heart Research Center Goettingen, University Medical Center Goettingen, University of Goettingen, Goettingen, Germany; Partner Site Lower Saxony, DZHK (German Centre for Cardiovascular Research), Goettingen, Germany; TORCH - Translational Registry of Cardiomyopathies, DZHK (German Centre for Cardiovascular Research), Berlin, Germany; Institute for Cardiomyopathies Heidelberg, University Hospital Heidelberg, Heidelberg, Germany; TORCH - Translational Registry of Cardiomyopathies, DZHK (German Centre for Cardiovascular Research), Berlin, Germany; Institute for Cardiomyopathies Heidelberg, University Hospital Heidelberg, Heidelberg, Germany; TORCH - Translational Registry of Cardiomyopathies, DZHK (German Centre for Cardiovascular Research), Berlin, Germany; Institute for Cardiomyopathies Heidelberg, University Hospital Heidelberg, Heidelberg, Germany; Department of Psychiatry and Medical Department, Section of Psychosomatic Medicine, Charité - Universitätsmedizin Berlin, Germany; Department of Cardiology and Pneumology, Heart Research Center Goettingen, University Medical Center Goettingen, University of Goettingen, Goettingen, Germany; Partner Site Lower Saxony, DZHK (German Centre for Cardiovascular Research), Goettingen, Germany; Cluster of Excellence “Multiscale Bioimaging: from Molecular Machines to Networks of Excitable Cells”, University of Goettingen, Goettingen, Germany; Core Unit for Bioinformatics (CUBI), Berlin Institute of Health (BIH) at Charité, Berlin, Germany; Deutsches Herzzentrum der Charité, Universitätsmedizin Berlin, Hindenburgdamm 30, 12203 Berlin, Germany; Deutsches Herzzentrum der Charité, Universitätsmedizin Berlin, Hindenburgdamm 30, 12203 Berlin, Germany; Berlin Institute of Health (BIH) at Charité—Universitätsmedizin Berlin, Anna-Louisa-Karsch-Straße 2, 10178 Berlin, Germany; Partner Site Berlin, DZHK (German Center for Cardiovascular Research), Potsdamer Straße 58, 10785 Berlin, Germany

**Keywords:** Inflammatory cardiomyopathy, Cytokines, Myocarditis, Pluripotent stem cells, Human aortic endothelial cells

## Abstract

**Aims:**

Inflammatory cardiomyopathy (iCMP) is a leading cause of heart failure, with limited therapeutic options. Excessive cytokine levels are implicated in adverse outcomes, but their pathomechanism in iCMP is poorly understood. We sought to identify key cytokines involved in severe iCMP and elucidate their potential contribution to cardiomyocyte injury.

**Methods and results:**

Cytokines were analysed in patients with severe biopsy-proven iCMP (*n* = 63; LVEF ≤ 35%) and validated in a national cohort (*n* = 425). *In vitro* experiments examined the effect of the top cytokines observed in severe iCMP with regards to production of reactive oxygen species (ROS) and calcium homeostasis in induced human pluripotent stem cell–derived cardiomyocytes (iPSC-CMs) and human aortic endothelial cells (HAECs). Three proteins (COLEC-12, CRIM-1, IL-6) were associated with severe iCMP. In iPSC-CMs, these proteins increased ROS (H2DCFDA assay) and intracellular Ca^2+^ levels (Fluo-4AM assay), indicating cellular stress. Effects were less pronounced in HAEC. Finally, real-world data from electronic medical records suggested a possible cardioprotective effect of clinically available inhibitors targeting these cytokines, although these findings remain exploratory and require confirmation in controlled studies.

**Conclusion:**

COLEC-12, CRIM-1, and IL-6 are elevated in severe iCMP and induce oxidative stress and calcium dysregulation in cultured cardiomyocytes. These findings highlight their potential as possible future therapeutic targets.

**Clinical Trial number:**

ClinicalTrials.gov Identifier: NCT04265040, NCT02187263

## Graphical Abstract

**Graphical Abstract:**
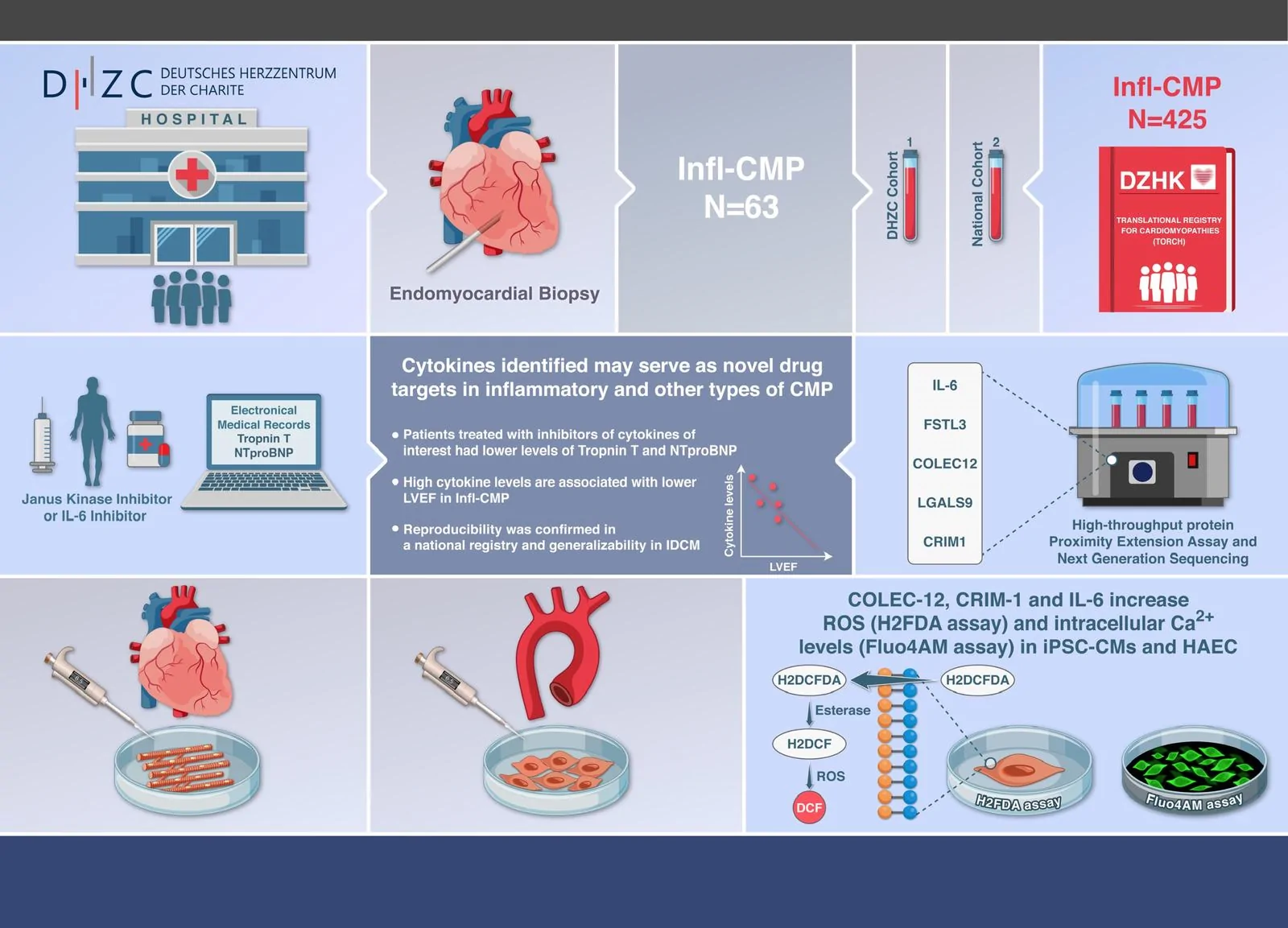

**Time of primary review: 42 days**

**See the editorial comment for this article ‘Inflammation meets calcium in iCMP’, by A. Ware**  ***et al*****., https://doi.org/10.1093/cvr/cvag108.**

## 1. Introduction

Inflammatory cardiomyopathy (iCMP) is one of the leading causes of sudden cardiac death (SCD) in young adults.^1^ Despite being a significant public health concern with potentially devastating outcomes,^2^ the pathophysiology of iCMP is not well understood, and the condition is often underdiagnosed,^3^ leading to poor treatment outcomes for many patients and high costs for the health care system.^4^

The development of drugs for iCMP has not much advanced for more than two decades,^5^ with steroids remaining a mainstay of therapy for severe cases.^6^ However, steroids are a non-specific treatment with potential serious side effects and consequently better therapies are needed.^6^ Despite the currently available treatments, a considerable number of patients initially diagnosed with complicated iCMP progress to chronic heart failure, enduring additional cardiovascular complications.^7^

A recent study by Chimenti *et al*.^8^ following patients with virus-negative chronic iCMP over 20 years found that patients treated with prednisone and azathioprine had better prognostic outcomes than the control group who was treated with standard heart failure therapy alone. Immunosuppressive therapy improved cardiovascular outcomes, conferring a significantly lower risk of cardiovascular death and heart transplantation, improvement in left ventricular ejection fraction (LVEF), and a lower likelihood of requiring an implantable cardioverter defibrillator.^8^ However, not all patients (12%) with virus-negative chronic iCMP respond to immunosuppression with prednisone and azathioprine.^8,9^

Current ongoing studies aim to better understand the pathophysiology and evaluate the effect of traditional and novel immunosuppressive therapies in iCMP in randomized, multicentre, double-blind, placebo-controlled trials (ARAMIS, Clinical Trials.gov: NCT 03018834; MYTHS, Clinical Trials.gov: NCT 05150704; IMPROVE-MC, ClinicalTrials.gov: NCT04654988; MAGiC-ART Clinical Trials.gov: NCT 04017936; CHASM-CS, Clinical Trials.gov: NCT 01477359).^3,10,11^

In recent years, there has been a growing interest in the role of inflammation in heart failure of various aetiologies. It is increasingly recognized that a systemic inflammatory state, particularly in iCMP may contribute to a decline in LVEF.^12–14^ The impact of systemic inflammation on cardiac injury and the role of cytokines has been increasingly raised in the literature.^15–17^ Therefore, we hypothesized that some cytokines in the context of iCMP may lead to myocardial injury and consequently to a decreased LVEF. Investigating cytokines that are associated with a severe clinical course of iCMP may help to identify novel drug targets that could be blocked with monoclonal antibodies^18,19^ or potentially removed through filtration or immunoadsorption^20,21^ in the future. Increasing evidence in the literature suggests that this is a reasonable approach, as inhibition of cytokines, their receptors, or intracellular kinases downstream of cytokine signalling was successfully applied in autoimmune diseases and other stages of heightened systemic inflammation such as iCMP,^4^ sepsis,^22^ COVID-19, and in cytokine storm after chimeric antigen receptor (CAR)-T cell treatment.^23^

In the present study, we have analysed two cohorts of patients. The study at our institution—DHZC (Deutsches Herzzentrum der Charité) consisted exclusively of patients with definite biopsy-proven iCMP. The national cohort also included patients who were diagnosed based on cardiac magnetic resonance imaging (CMR).^24^ Patients were divided into a group with severe iCMP (LVEF ≤ 35%) and patients with mild-to-moderate iCMP (LVEF > 35%). The choice for a cut off at 35% for LVEF was guided by the European Society of Cardiology (ESC) guidelines,^25^ in which this cut off is applied as an indicator for clinical severity and risk of severe arrhythmias.

The identified proteins were subsequently tested in cell culture with induced human pluripotent stem cell-derived cardiomyocytes (iPSC-derived CM) and human aortic endothelial cells (HAEC) to evaluate for a potential harmful effect on cells vs. the possibility that these proteins are merely bystanders in the inflammatory cascade.

Finally, in order to investigate potential associations between the cytokines we identified and iCMP, we conducted an exploratory analysis examining whether therapeutic inhibitors of these cytokines used in clinical practice were associated with reductions in blood markers of cardiac injury, suggesting possible cardioprotective effects. For that purpose, we tested our hypothesis using real-world data extracted from electronic medical records (EMRs) of patients who received cytokine inhibitors for various indications at our institution.

## 2. Methods

### 2.1 Study participants and recruitment

We investigated samples from 63 patients collected in the biobank of the DHZC, Campus Benjamin Franklin, Berlin between 2014 and 2021. Sample collection took place at the time of diagnosis, when patients presented with acute symptoms of inflammatory cardiomyopathy. Endomyocardial biopsies (EMBs) were obtained during routine diagnostic workup as indicated by the ESC Consensus Statement^26^ and American Heart Association Guidelines.^27^ As recommended, EMBs were obtained in clinical scenarios, in which the results of histology were expected to change clinical treatment. Coronary artery disease was excluded in all patients by coronary angiography. Blood samples were systematically collected in EDTA plasma tubes, processed, and stored at −80°C at the time of EMB.

Based on the results of histology and comprehensive immunohistochemistry of EMBs, we identified patients with iCMP (*n* = 63) according to the definition of the ESC.^26^

Patients taking immunosuppressive or anti-inflammatory therapy at the time of EMB were excluded to avoid potential confounders.

Based on echocardiographic data, patients were further divided into groups with severe iCMP (LVEF ≤ 35%) and those with mild-moderate iCMP (LVEF > 35%).

### 2.2 Validation of results in an independent national cohort

To validate our initial results in an independent cohort, we investigated blood samples from patients with iCMP collected in a German national registry for cardiomyopathies. This registry contained samples from patients with a diagnosis of iCMP collected within the Translational registry for cardiomyopathies (TORCH) network of the German Center for Cardiovascular Research e.V. (eingetragener Verein)/Deutsches Zentrum für Herzkreislaufforschung (DZHK).

Patients were recruited at cardiovascular centres across Germany within the DZHK network since 2014. Samples were collected in EDTA tubes and EDTA plasma was extracted and stored within 1 h after collection at −80°C.

Similar to the DHZC cohort, patients who had received immunosuppression prior to sample collection were excluded from the analyses. Furthermore, patients missing relevant clinical data were excluded. Samples that qualified for our study were derived from 11 German centres. In this independent cohort, 354 patients were diagnosed based on EMB and 71 patients based on CMR. Modified Lake Louis criteria were used to diagnose iCMP by CMR.^28^

### 2.3 Cytokine analysis

For cytokine analysis, EDTA plasma from patients was analysed with the Proximity Extension Assay (PEA) by Olink. In brief, the method is based on the binding of proteins by a pair of antibodies that carry unique complementary oligonucleotide sequences. When two complementary oligonucleotide sequences come to proximity, they hybridize and can be extended by a DNA polymerase to form a complementary strand. The DNA amplicon can then be quantified using real-time PCR or next generation sequencing. A major advantage of PEA technology over traditional antibody-based assays is enabling biomarker analysis with high multiplexing as well as with high specificity and throughput.^24^

Olink's Explore 384—‘Inflammation’ subpanel, a part of the Explore 3072 panel, was used for our analyses. The full list of proteins included into the Olink Explore 384—Inflammation panel is provided in the Supplementary material online, *File S1* ‘Olink 384 Inflammation list’.

The preparation of the sample libraries, randomization, quality control, and data processing took place in a process standardized by Olink (for more information, please see the website https://www.olink.com). Data are presented as normalized protein expression (NPX), providing relative quantification between samples, since calibration to absolute concentrations was not conducted for individual samples or assay batches. Log-transformed values were used to obtain a normal distribution.

### 2.4 Statistical analysis of patient samples

Statistical analysis was conducted using R. The two batches of Olink data were integrated using the olink_normalization function from the OlinkAnalyze package (v. 3.1) and 16 bridging samples were present in both data sets. Cytokine level association with LVEF was analysed with a linear regression model (R function lm), using the normalized protein levels (NPX) as the predictor variable and LVEF as the response variable, with cohort (DHZC/national cohort) and patient sex and age as covariates. *P*-values were corrected for multiple testing using a false discovery rate (FDR) controlling procedure (Benjamini–Hochberg method).^29^ Cytokines with an FDR <0.05 were considered statistically significant. R markdown documents and scripts were used to generate all results and figures in this manuscript and Supplementary Data are available from https://github.com/bihealth/manuscript-myocarditis/.

### 2.5 Cardiac differentiation of iPSCs, quality control, and maintenance of iPSC-derived CMs

All protocols for studies with iPSC were approved by the Goettingen University Ethical Board (15/2/20, 20/9/16An, and 7/5/24) and the Odense University Ethical Board (Projekt ID S-20140073HLP). Informed consent was obtained from all participants, and all research was performed in accordance with relevant guidelines and regulations. Induced human pluripotent stem cells (iPSCs) were described previously ^30,31^ and were a kind gift from Joseph C. Wu (Stanford University, CA). Adaptions of that protocol and characterization are reported by Lian *et al*.^32^ and Ebert *et al*.^33^ Human iPSCs were grown on matrigel-coated plates (ES qualified, BD Biosciences) using chemically defined E8 medium and passaged every 3–4 days using EDTA (Thermo Fisher, 15575020) as described previously.^33,34^

Human iPSCs were cultured and differentiated into iPSC-derived CMs as described previously^32,35,36^ using a small molecule-based differentiation protocol reported before.^32,35^ From day 7 of cardiac differentiation, spontaneously contracting iPSC-derived CMs were observed. Human iPSC-derived CMs were cultured in RPMI medium (Life Technologies, 11875093) complemented with B27 supplement (Life Technologies, 17504044). On day 20–25 of cardiac differentiation, iPSC-derived CMs were dissociated with TrypLE (Life Technologies, 12604013) and used for the respective experimental analysis. The 96-well plates with a cell density of 10 thousand cells/well and the 12-well plates with a cellular density of 250 thousand cells/well were then maintained for a resting period of 24 h. The differentiation process and maintenance, as well as storage during protein exposure or incubation time of the assays, which should be performed in the incubator, was done at 37°C and 5% CO_2_.

Immunofluorescence staining and confocal imaging were performed to validate cardiomyocyte differentiation of human iPSC-derived cells. Cells were cultured on glass coverslips (Thermo Fisher Scientific), fixed with 4% paraformaldehyde (PFA), and permeabilized using 0.2% Triton X-100 (Thermo Fisher Scientific) at room temperature. After blocking with appropriate blocking buffer, samples were incubated overnight at 4°C with primary antibodies against cardiac troponin T (TNNT2, Thermo Fisher Scientific), sarcomeric alpha actinin (SAA, Sigma), and myosin-binding protein C (MYBPC3, Abcam). Following washing steps, Alexa Fluor–conjugated secondary antibodies (Thermo Fisher Scientific) were applied at room temperature.

Coverslips were mounted onto glass slides using antifade mounting medium and imaged using a confocal laser scanning microscope (LSM 710 Meta, Zeiss) equipped with a 63× oil-immersion objective, as previously described.^31,37,38^ The results are displayed in Supplementary material online, *Figure S1*.

Additionally, reverse transcriptase real-time polymerase chain reaction (RT-PCR) was performed to validate cardiomyocyte-specific gene expression in iPSC-derived cardiomyocytes. Total RNA was isolated using the miRNeasy Micro Kit (Qiagen; 217084) according to the manufacturer’s instructions, followed by DNase treatment (Thermo Fisher Scientific; AM1907) to eliminate genomic DNA contamination. For cDNA synthesis, 500 ng of total RNA was used.

RT-PCR was conducted using TaqMan Gene Expression Assays and TaqMan Gene Expression Master Mix (Applied Biosystems; 4369016) on an Abbott StepOnePlus RT-PCR system. The following cardiomyocyte marker genes were analysed: TNNT2 (Hs00943911_m1), MYH2 (Hs00166405_m1), MYH7 (Hs01110632_m1), TNNI3 (Hs00165957_m1), and ACTC1 (Hs01109515_m1). Eukaryotic 18S rRNA (Hs03003631_g1) was used as the housekeeping gene. Relative gene expression was calculated using normalization to the housekeeping gene, and all reactions were performed in technical duplicates (HAEC) and quadruplicates (iPSC-CM). The results are displayed in Supplementary material online, *Figure S2*.

### 2.6 Preparation of primary HAECs

Primary HAECs were obtained from Cell Applications (Cat. No. 304-05a, Lot:2139) as a frozen stock stored in liquid nitrogen. The cells were thawed and transferred with Endothelial Cell Basal Medium (PromoCell, C-22211), Supplement Pack (PromoCell, C-39211) + 10% foetal bovine serum (Pan-Biotech, P40-47500) + 1% penicillin/streptomycin (Fa Sigma, 4049) to T-75 flasks. Medium was changed every 48 h. The 6th passage was plated on uncoated 96- and 12-well plates. Plating and maintenance conditions were the same as described for the cardiomyocytes above.

### 2.7 Protein exposure

After the described resting period, proteins were added to the HAEC and CM wells. Protein concentrations used for cell exposure experiments were derived from NPX values and converted to approximate absolute concentrations based on Olink’s reference calibration curves to guide experimental dosing. The concentrations represent the measured mean values from the cohort of patients with reduced LVEF and severe iCMP, IL-6 (8.6 pg/mL, 206-IL Biotechne), CRIM-1 (1000 pg/mL, 1917-CM-050 Biotechne), and COLEC-12 (6400 pg/mL, 2690-CL-050 Biotechne) dissolved in PBS + 0.1% BSA (Gibco, 14190-144 and Pan-Biotech, P40-47500). To the control wells, the same amount of pure PBS + BSA was added. The timer was started, and analyses were performed after 24 and 48 h.

### 2.8 Quantitative ROS production analysis using the H2DCFDA assay

Intracellular ROS generation was assessed using the H2DCFDA Kit (D399, Thermo Fisher) according to the manufacturer’s instructions. The H2DCFDA working solution was prepared in PBS (70011044, Thermo Fisher) at a final concentration of 5 µM. Cells were incubated with the dye for 30 min at 37°C and 5% CO_2_, followed by two wash cycles with PBS.

All applied solutions were analysed separately to control for background fluorescence. As a positive control, 10 µL of the H2O2 solution (final concentration 1.29 M) was added. Fluorescence intensity was measured using a CLARIOstar Plus plate reader.

### 2.9 Qualitative calcium in- and efflux analysis with fluo-4AM

The change in intracellular calcium homeostasis was measured using the Fluo-4 AM Kit (F14201, Thermo Fisher). The assay was performed as recommended by the manufacturer. HAECs and CMs were stained with 100 µM Fluo-4 working solution (Fluo-4, AM/Probenecide c = 5 µM, c = 1.5 mM) and incubated for 30 min at 37°C and 5% CO_2_. Two wash cycles were included with Tyrode Buffer (Thermo Scientific, J67607.K2). All used solutions were analysed separately to test for individual fluorescence. As a positive control, 1 M potassium chloride was used. The visual evaluation of the fluorescence was performed with a KEYENCE BZ-9000 microscope.

### 2.10 Plate reading and viability evaluation

For plate reading, a CLARIOstar Plus plate reader (BMG Labtech) was used. For daily evaluation of the cell culture and assessment of confluence and viability, a Zeiss Axiovert 25 microscope was used. Pictures of the cells were obtained using a KEYENCE BZ-9000 microscope.

### 2.11 Evaluation for potential correlation of cytokines in EMR real-world data

To further test our hypothesis that elevation of specific cytokines can be harmful to the heart, in an exploratory analysis, we investigated laboratory markers of cardiac injury in patients before and after treatment with cytokine inhibitors. These data were collected from three rheumatology departments at the Charité Universitätsmedizin, Berlin. EMR data were extracted from the clinical information system for the period from January 2018 to May 2023. The analysis concentrated on patients who were treated with inhibitors specifically targeting the cytokines identified in our study or the pathways these cytokines influence downstream.

This encompassed patients who had been administered IL-6 or Janus kinase inhibitors and for whom high-sensitivity troponin T (hs-TnT) and N-terminal prohormone of brain natriuretic peptide (NT-proBNP) measurements were obtainable. A matched cohort (based on age and sex) and a paired samples approach were used to compare laboratory parameters before and after initiation of cytokine inhibitor therapy. For the matched approach, only laboratory values obtained after the initiation of cytokine inhibitors were included in the analysis. From these laboratory values, the median was calculated for each patient. For patients who did not receive any cytokine inhibitors and therefore belonged to the control group of this analysis, the median was calculated from all available timepoints.

For each cytokine investigated, a control group was established at a 1:4 ratio using the nearest matching procedure, identifying comparable individuals who had not undergone treatment with cytokine inhibitors.

Matching criteria included age, sex, renal insufficiency, cardiac, rheumatologic, and autoimmune comorbidities. Patients with a glomerular filtration rate (GFR) below 30 mL/min/m^2^ were excluded from the analysis, as hs-TnT and NT-proBNP are affected by renal function. Extreme outliers were identified and reduced using the winsorizing method.^33^ Subsequently, the medians common logarithms were compared using a Mann–Whitney *U* test and Tukey’s Honest Significant Difference test.

For the paired samples approach, patients were identified for whom laboratory parameters were available both before and after initiation of cytokine inhibitor treatment. The common logarithm of the difference between median values for both time points was calculated and compared using the one sample Wilcoxon test against zero in null hypothesis.

Additionally, a generalized linear model was applied, utilizing the same criteria as in the matching approach. Again, patients with a GFR below 30 mL/min/m^2^ were excluded from this analysis.

This analysis was conducted to provide an exploratory and preliminary assessment of potential associations indicative of cardiac injury, as reflected by hs-TnT and NT-proBNP measurements.

### 2.12 Ethics committee approval

This study was reviewed and approved by the ethics committee of DHZC Universitätsmedizin Berlin (EA4/056/20, EA1/187/22) and the Ethikkommission Medizinische Fakultät Heidelberg (S-344/2014) and was conducted in accordance with the Declaration of Helsinki. All patients provided their written informed consent.

In the TORCH registry, all participants provided written informed consent, and the study received approval from the ethics committees of the participating study centres. Additionally, a peripheral blood sample was collected from each participant. Standard operating procedure for quality training and monitoring was regularly conducted, following the methodology described by Schwaneberg *et al*. (2017).

The data were analysed by Phillip Suwalski, Ainoosh Golpour, January Weiner, Nicolas Musigk, and Bettina Heidecker.

## 3. Results

### 3.1 Study population

We enrolled a total of 488 patients with iCMP (*Table 1*) and analysed clinical data of a total of 836 patients. The DHZC cohort was composed of 63 patients who were recruited from the DHZC, while the national cohort consisted of 425 patients who were enrolled within the TORCH registry of the National Cohort (DZHK). In addition, we investigated EMR data from 348 patients.

**Table 1:** Demographic data of Deutsches Herzzentrum der Charité (DHZC) cohort and National cohort

Cohort	DHZC cohort (*n* = 63)	National cohort (*n* = 425)	*P*-value
Registry	DHZC	DZHK	
Sex female (*n*, %)	19 (30%)	120 (28%)	0.87
Age in years (median, IQR)	54 (36–62)	49 (37–58)	0.12
BMI in kg/m^2^ (median, IQR)	27 (25–29)	26 (24–30)	0.95
LVEF ≤ 35% (*n*, %)	23 (37%)	169 (40%)	0.72
LVEF (median, IQR)	45 (31–58)	42 (30–55)	0.23
eGFR in mL/min/1.73 m^2^ (median, IQR)	78 (69–99)	87 (70–103)	0.32

The *P*-values were calculated using a χ2 test for categorical variables (sex and LVEF ≤ 35%) and a Wilcoxon test for continuous variables (age, BMI, LVEF, eGFR).

There were no significant differences among the groups in key baseline characteristics such as sex, age, body mass index (BMI), LVEF, and renal function. Women comprised 28 to 30% of the participants across both cohorts. The median age of the participants spanned from 49 to 54 years. The median BMI was approximately 26–27 kg/m^2^, defining the participants, on average, as overweight.

The two groups were categorized based on the extent of reduction in LVEF, with patients classified as having either severe LVEF reduction (≤35%) or mild-to-moderate LVEF reduction (>35%). The median LVEF was in the range of 42–45%. The median eGFR was 78 mL/min/1.73 m^2^ in the DHZC cohort and 87 mL/min/1.73 m^2^ in the national cohort (*P* = 0.32).

### 3.2 Cytokine analysis in the DHZC cohort

First, we tested the hypothesis that elevation of some cytokines in the blood of patients with iCMP may lead to myocardial injury and consequently to decreased LVEF. A linear regression model was employed to analyse the relationship between cytokine levels and LVEF in patients with iCMP.

After correction for multiple testing, five cytokines were found to be significantly associated with LVEF in the DHZC cohort of patients with iCMP (DHZC, *n* = 63) [FDR < 0.05; Supplementary material online, *Table S1*; Supplementary material online, *File S2* (full_results.xlsx)]. The relationship between relative cytokine concentration and LVEF was inverse, with higher relative cytokine concentrations associated with lower LVEF in patients with iCMP.

### 3.3 Replication of findings in an independent national cohort

To evaluate for robustness of the results obtained from the DHZC cohort, we utilized patients diagnosed with iCMP from the TORCH registry of the DZHK. Sixteen bridging samples were used to combine the data set from our DHZC cohort (*n* = 63) with the TORCH registry samples (*n* = 435). The normalization procedure followed the recommendations of the manufacturer.

The data were normalized using the OlinkAnalize package, version 3.1.0.

Results of the normalization process are presented in Supplementary material online, *Figures S3* and *S4*.

Principal component analysis was performed to ensure that no subgroups differentiated the DHZC and national datasets. No significant differences were observed for the first and second principal components between the datasets. As such, the datasets were considered comparable (see Supplementary material online, *Figure S5* and Supplementary material online, *Figure S6*).

Then, a linear regression model was employed to analyse the relationship between cytokine levels and LVEF in the independent national cohort of patients with iCMP collected in the national registry (TORCH, *n* = 425). Following correction for multiple testing, four out of five of the identified cytokines from the DHZC cohort were replicated in the national cohort. COLEC-12, ranked as the 6th most associated cytokine with LVEF in the DHZC cohort (*P*-value: 2.19×10^−5^, FDR: 0.08), emerged as the top-ranked cytokine in the national cohort (*P*-value: 4.46×10^−16^, FDR: 1.64×10^−13^). Overall, there were 77 cytokines significantly associated with LVEF [FDR < 0.05; Supplementary material online, *Tables S2* and S3; Supplementary material online, *File S2* (full_results.xlsx)].

The relationship between relative cytokine concentration and LVEF was again inverse (*Figure 1*). Data from the national cohort demonstrated that the cytokines most strongly associated with severe iCMP in the DHZC cohort were replicated in iCMP in the independent national cohort (*Table 2*; complete list available in Supplementary material online, *File S1* full_results.xlsx). There were no significant differences in the expression of the presented cytokines based on age or sex differences (data not shown). Future studies will aim to perform sex-based cytokine profiling to investigate potential sex specific differences in iCMP.

**Figure 1:**
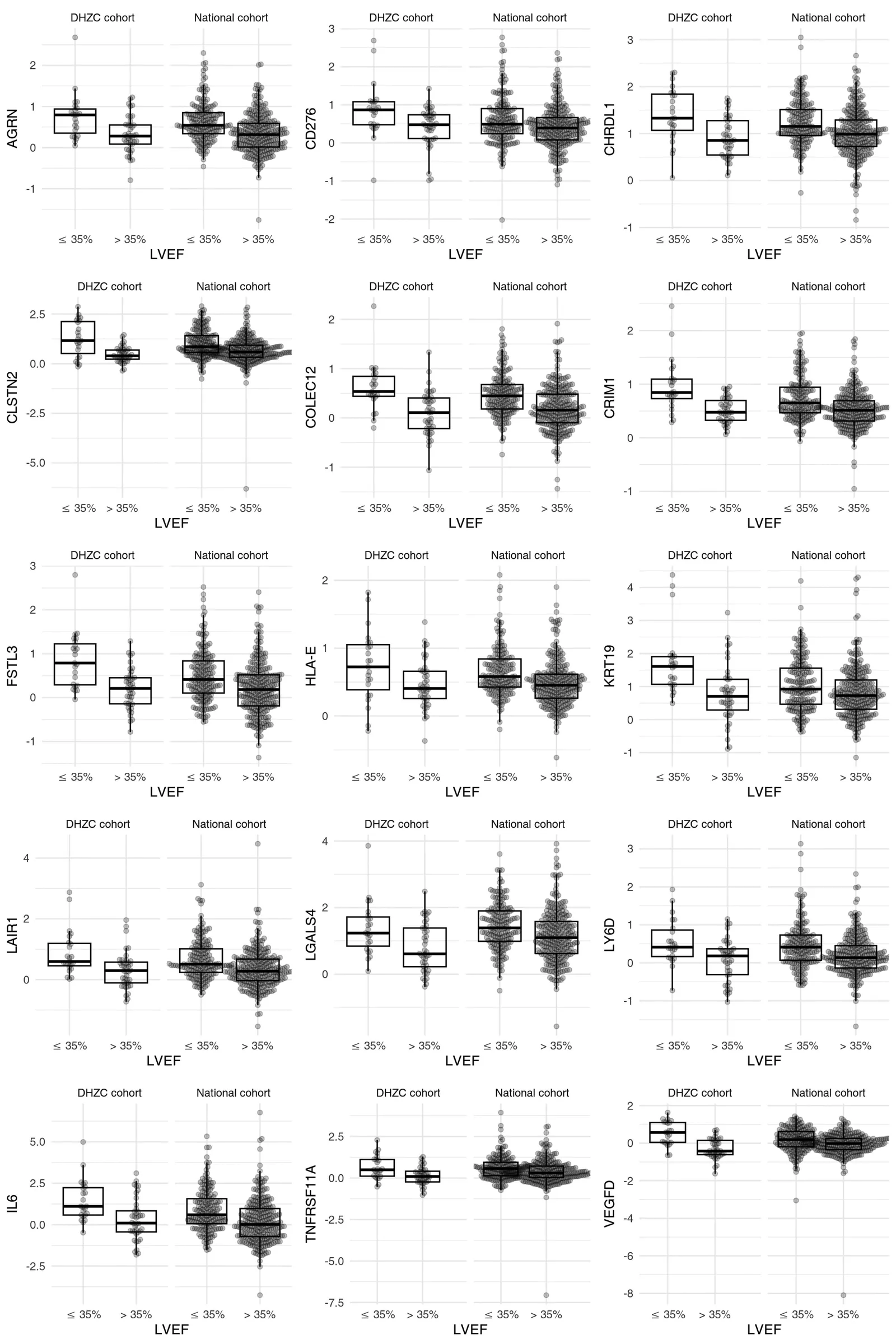
Relative cytokine concentrations in patients with inflammatory cardiomyopathy in both DHZC and national cohorts: the patients (*n* = 488) were grouped according to cohort and left ventricular ejection fraction (LVEF). The selected cytokines are the top cytokines identified in the DHZC cohort. Their association with LVEF was significant in both cohorts combined (DHCZ and national cohort, FDR < 0.001). FDR was calculated using the Benjamini–Hochberg procedure from the *P*-value derived from a linear regression model). For this analysis, one patient sample was included, with each sample analysed in duplicate to ensure technical consistency.

**Table 2:** Top 10 cytokines associated with low left ventricular ejection fraction (LVEF): cytokines were derived from the Deutsches Herzzentrum der Charité and the national cohort (*n* = 488)

Cytokine	Estimate	r2	*P* value	FDR
COLEC12	−12.58	0.1762	<2×10^−16^	<2×10^−16^
PLAUR	−11.25	0.163	<2×10^−16^	4.2×10^−15^
AGRN	−11.2	0.1549	<2×10^−16^	4.6×10^−14^
WNT9A	−10.03	0.1492	6.5×10^−16^	2.4×10^−13^
LAIR1	−8.063	0.144	2.9×10^−15^	1.0×10^−12^
LILRB4	−8.768	0.1439	3.0×10^−15^	1.1×10^−12^
FABP1	−4.191	0.1416	5.8×10^−15^	2.1×10^−12^
CRIM1	−13.45	0.1403	8.4×10^−15^	3.0×10^−12^
CCL3	−6.865	0.1389	1.3×10^−14^	4.5×10^−12^
FSTL3	−8.187	0.1324	8.1×10^−14^	2.9×10^−11^

Higher relative cytokine concentrations were associated with lower LVEF values. Estimate, the coefficient estimate from the linear regression model; r^2^, coefficient of determination (r^2^) value for the model; *P*-value, raw *P*-value for the model; FDR, false discovery rate calculated using Benjamini–Hochberg correction.

### 3.4 *In vitro* effects of selected cytokines on iPSC-derived cardiomyocytes (iPSC-derived CM) and HAECs

#### 3.4.1 Analysis of ROS production using the H2DCFDA assay in iPSC-derived cardiomyocytes

COLEC-12, CRIM-1, and IL-6 were selected for functional testing in iPSC-derived CM and HAEC given their highly elevated blood levels in patients with severe iCMP and their biological plausibility based on prior literature,^39–45^ which reported their involvement in inflammation,^4,12,17,46^ oxidative stress,^39,41,47^ and tissue remodelling.^4^ An overview of the experimental workflow is provided in Supplementary material online, *Figure S7*. The cardiomyocyte identity of iPSC-CMs was experimentally validated by immunostaining and RT-PCR analysis of cardiomyocyte-specific marker genes (see Methods and Supplementary material online, *Figures S1* and *S2*).

COLEC-12 is a scavenger receptor involved in immune defence, previously described as playing a role in apoptosis regulation and inflammation in neoplastic diseases.^41^ Additionally, it is associated with regulatory functions in pattern recognition and activation of the innate immune system.^48^ Pattern recognition plays a key role in viral myocarditis by inducing molecular mimicry, a well-known mechanism in its development.

CRIM-1 has been linked to congenital heart disease, suggesting a potential inherited risk factor.^49^ It is also associated with worsening chronic heart failure and cardiac fibrosis in human hearts.^38,50^

In addition, we tested the effect of IL-6 on those cell lines. IL-6 was among the top cytokines associated with severe iCMP in our cohorts (FDR 6, 13×10^−6^) and has been reported as a potentially critical factor in the development and exacerbation of severe iCMP.^4^ The IL-6-mediated transition of monocytes into potentially harmful macrophages is considered a key component in the pathogenesis of myocarditis as suggested by multiple studies.^43–45,51,52^ Given the well-established role of IL-6 in inflammatory cardiovascular disease and the availability of clinically approved inhibitors of IL-6, we included IL-6 among our primary candidates for mechanistic testing. The exact mechanisms of the IL-6 pathway in iCMP and its potential as a therapeutic target are still under investigation.^4^ IL-6 is of particular interest, given that FDA-approved inhibitors including Tocilizumab or Sarilumab are already available and have been used as therapy in COVID-19.

Supplementary material online, *Figure S8* illustrates ROS production in human iPSC-derived CMs after exposure to COLEC-12, CRIM-1, or IL-6. The cytokine concentration used corresponded to the mean concentration of the individual cytokine in our cohort of severe iCMP. Each individual cytokine induced a significant increase in ROS production in iPSC-derived CMs after 24 and 48 h of exposure compared to the untreated control (baseline). COLEC-12 induced the strongest ROS response, showing an 8.5-fold (standard deviation, SD 0.45) increase compared to control levels after 24 h, with a sustained elevation to 6.1-fold (SD 0.76) at 48 h. IL-6 followed, exhibiting a similar response with an 8.3-fold (SD 0.50) increase at 24 h, reaching 8.7-fold (SD 1.48) within the first 48 h. In contrast, CRIM-1 induced a peak increase of 7.0-fold (SD 2.01) at 24 h, which declined to 6.5-fold (SD 0.66) after 48 h. *P* values are listed in Supplementary material online, *Table S4*.

#### 3.4.2 Analysis of ROS production using the H2DCFDA assay in human aortic endothelial cells

Supplementary material online, *Figure S9* illustrates ROS production in HAEC in response to cytokine type and exposure duration, following the same experimental conditions as in the iPSC-derived CM assays. All cytokines showed a significant ROS increase after 24 and 48 h of exposure, compared to the untreated control (baseline). IL-6 elicited the most substantial ROS response, with an 8.9-fold (SD 0.63) increase compared to controls after 24 h, which decreased to 3.04-fold (SD 0.28) at 48 h. COLEC-12 led to a 4.1-fold (SD 0.12) increase at 24 h, declining to 3.0-fold (SD 0.76) at 48 h. CRIM-1 induced a peak 3.9-fold (SD 0.50) increase at 24 h, which reduced to 3.2-fold (SD 1.06) after 48 h. *P*-values are listed in Supplementary material online, *Table S5*.

#### 3.4.3 Analysis of calcium uptake using the fluo-4AM assay in iPSC-derived cardiomyocytes

*Figure 2* presents the effects of COLEC-12, CRIM-1, and IL-6 exposure of iPSC-derived CMs over time on the cellular calcium uptake on representative samples. The experiments were repeated twice independent of each other, and results were replicated. Results were compared to negative and positive controls. The positive control, treated with potassium chloride, consistently indicates cellular stress through elevated calcium uptake (green fluorescence). In the first 24 h, a mild response was observed in iPSC-derived CMs treated with any of the three cytokines. However, iPSC-derived CMs treated with COLEC-12 and CRIM-1 develop intense fluorescent activity after 48 h, while iPSC-derived CMs treated with IL-6 appear to return to baseline levels comparable to the negative control.

**Figure 2:**
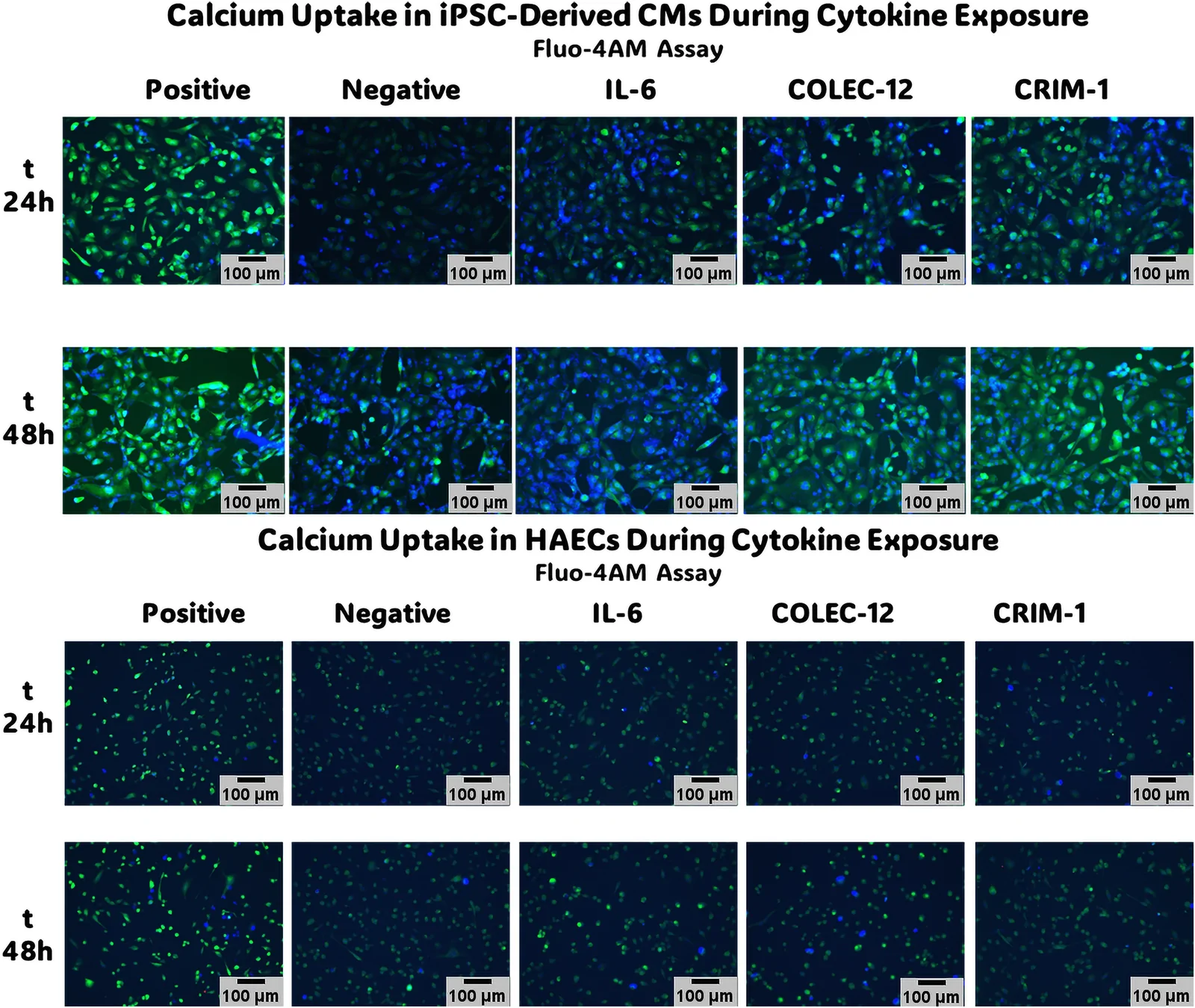
Calcium uptake in iPSC-derived CMs and HAECs during cytokine exposure: results of the intracellular calcium measurement of induced human pluripotent stem cell-derived cardiomyocytes (iPSC-derived CMs) and human aortic endothelial cells (HAECs) exposed to COLEC-12, CRIM-1, or IL-6 for 24 and 48 h. The cells were stained with the Fluo-4 AM assay (green fluorescence) and Hoechst indicator (blue fluorescence). Potassium chloride was used as a positive control. The scale bar represents 100 µm. The Fluo-4 AM assay was conducted in two independent experiments using the same cell lines.

#### 3.4.4 Analysis of calcium uptake using the fluo-4AM assay in HAEC

*Figure 2* illustrates the effect of cytokine exposure duration on calcium homeostasis in HAECs on representative slides. The experiments were repeated twice independent of each other, and the results were replicated. Results were compared to negative and positive controls. While the positive control demonstrates expected cellular response, no distinct visual enhancements are observed across cytokine treatments within the first 24 h. After 48 h, IL-6 and COLEC-12 demonstrate slight response, indicating a delayed reaction to cytokine exposure.

### 3.5 Exploratory EMR data evaluation

We screened 18 566 patient records from three rheumatology departments of the Charité University Berlin for an exploratory analysis of EMR data. Among these, hs-TnT and/or NT-proBNP measurements were available for 8767 patients and served as surrogate markers of cardiac injury. Within this patient pool, 348 individuals had been administered with specific inhibitors for lead cytokines of this study for various rheumatological diseases. These included IL-6 inhibitors and Janus kinase inhibitors.

For other cytokines identified in our study, there are either no specific inhibitors presently in clinical use, or the number of patients treated was insufficient to permit robust statistical analysis.

#### 3.5.1 NT-proBNP

Treatment with IL-6 receptor inhibitors resulted in a notable reduction in median NT-proBNP levels by 216 ng/L (*n* = 76; *P* < 0.001) for the matched cohort. The comparison of values of paired samples before and after initiation of cytokine inhibitor therapy (*n* = 40) supports this finding; a significant NT-proBNP reduction of 95 ng/L (*P* = 0.005) was detected. The adjusted *P*-value for age, sex, renal insufficiency, heart failure, rheumatologic, and autoimmune comorbidities was highly significant (*P* < 0.001).

Similarly, in the group receiving Janus kinase inhibition (*n* = 97) the median NT-proBNP level was significantly reduced by approximately 216 ng/L (*P* < 0.001) for the matched cohort. The comparison of NT-proBNP levels of paired samples before and after initiation of cytokine inhibitor therapy (*n* = 44) supports this finding with a significant reduction of NT-proBNP by 45 ng/L (*P* = 0.045). The adjusted *P*-value for age, sex, renal insufficiency, cardiac, rheumatologic, and autoimmune comorbidities remained significant (*P* = 0.016). The results are presented in *Figure 3A*–3F).

**Figure 3:**
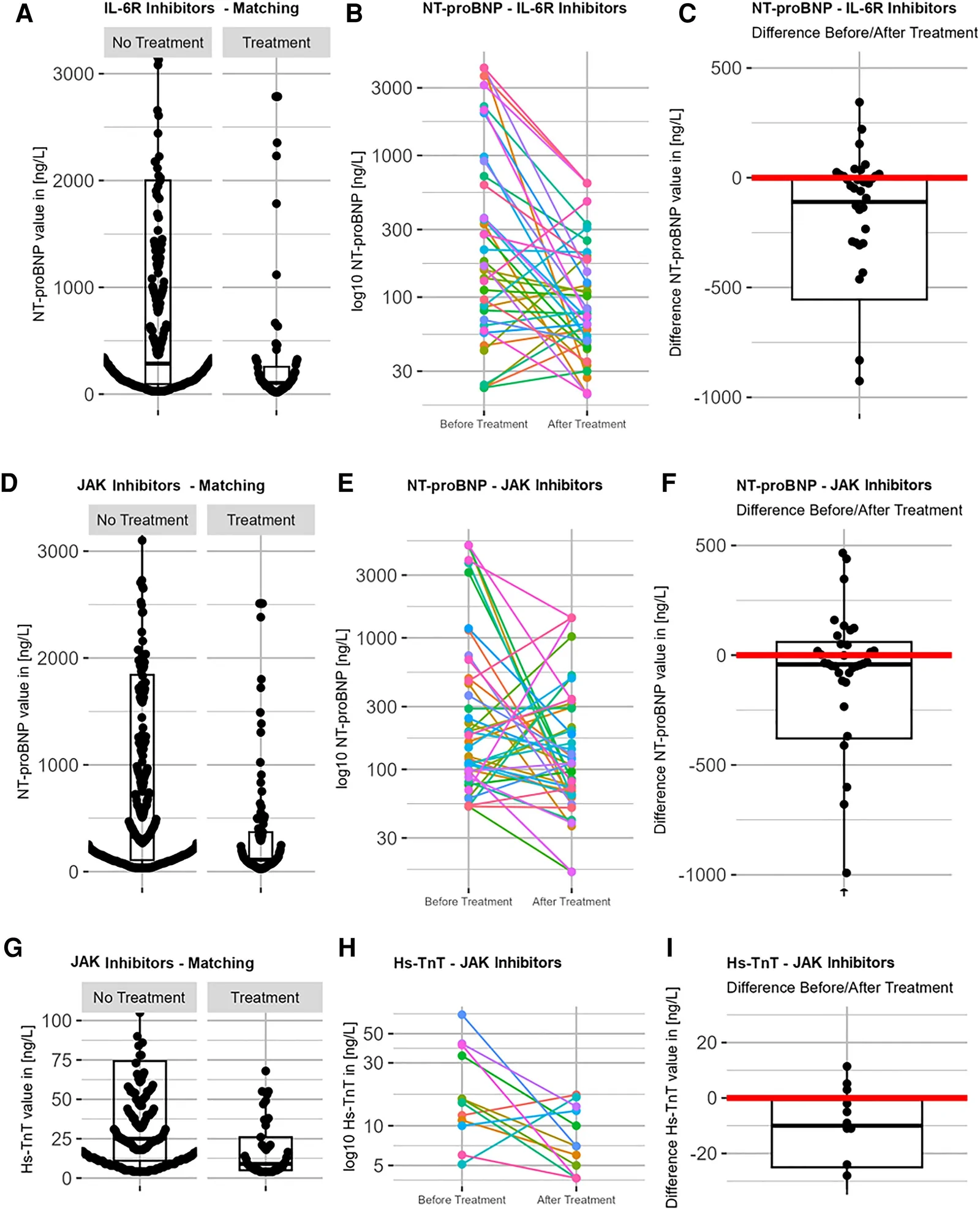
Comparison of NT-proBNP or Hs-TnT between patients with IL-6 or Janus kinase inhibitors and matched controls: comparison of N-terminal prohormone of brain natriuretic peptide (NT-proBNP) or high-sensitivity troponin T (Hs-TnT) values in ng/L between patients who were prescribed IL-6 receptor inhibitors (NT-proBNP *n* = 40) or Janus kinase (JAK, Hs-TnT *n* = 53, NT-proBNP *n* = 97) inhibitors with matched controls not taking cytokine inhibitors (A, D, and E). Comparison of NT-proBNP (IL-6 receptor inhibitor *n* = 40 and JAK inhibitor *n* = 44) or Hs-TnT (*n* = 12) values in paired samples before and after initiation of cytokine inhibitors (B, E, and H) and visualization of the difference of cytokine levels before and after initiation of cytokine inhibitors (C, F, and I). In A and G, a one-sample Wilcoxon test against zero in the null hypothesis was performed. In H, the medians of the common logarithms were compared using the Mann–Whitney *U* test. The presented data were obtained from the electronic medical records of the patients and measured once before and after treatment by the accredited hospital laboratory according to standardized procedures.

#### 3.5.2 Hs-TnT

In the group receiving Janus kinase inhibitors, a significant decrease in median hs-TnT levels by 16 ng/L (*n* = 53; *P* < 0.001) was detectable for the matched cohort. The comparison of levels in paired samples before and after initiation of cytokine inhibitors (*n* = 12) supports this finding with a significant reduction of hs-TnT (*P* = 0.028) by 8.5 ng/L. The adjusted *P*-value for age, sex, renal insufficiency, heart failure, rheumatologic, and autoimmune comorbidities remained significant (*P* = 0.023). The results are presented in *Figure 3G*–3I).

## 4. Discussion

This study addressed an important knowledge gap in the understanding of iCMP, by investigating cytokines that might represent potential therapeutic targets in patients who do not typically respond to existing, advanced therapies. We hypothesized that some cytokines released in the context of severe iCMP may contribute to myocardial injury and result in reduced LVEF. Therefore, we investigated the relationship between cytokines and LVEF. Our findings supported our hypothesis that higher levels of specific cytokines were correlated with greater reductions in LVEF.

We first derived cytokine associations with iCMP in a cohort from our institution (*n* = 63). We then successfully replicated our findings in an independent cohort (*n* = 425), which encompassed samples from 11 different cardiovascular centres across Germany, which belong to the national TORCH registry.

Both the DHZC and the national cohort demonstrated a comparable distribution of key baseline parameters including sex, age, BMI, LVEF, and renal function. The sex distribution, with approximately one-third of the participants being women agrees with prior literature.^53^ Based on the median BMI, most patients were overweight, an observation that aligns with findings from prior work on iCMP.^54^ One may speculate that proinflammatory characteristics of adipose tissue could contribute to a predisposition for developing iCMP.^54^

A considerable proportion of patients balanced between the two groups (36.5% in the DHZC cohort and 39.8% in the national cohort) exhibited severely compromised left ventricular function (LVEF ≤ 35%). Kidney function was overall preserved in both cohorts.

According to ESC guidelines,^25^ patients with LVEF ≤35% received more frequently angiotensin-receptor blockers (ARBs) and diuretics compared with those with a higher LVEF. If this disparity in medication regimen influenced our study outcome, it likely diminished the observed association between specific cytokines and reductions in LVEF, given that ARBs have anti-inflammatory properties.^46,55^

Several of the cytokines identified had been proposed or evaluated as potential therapeutic targets in iCMP previously,^43–45,52^ supporting our results on a larger scale.

Similar to prior studies in patients with heart failure overall,^43–45,52^ higher serum levels of IL-6 were associated with more severe forms of iCMP in our study. Importantly, it had been shown previously that in IL-6-deficient mice, prevalence and severity of experimental myocarditis were reduced in the absence of IL-6.^52^ In myocarditis, IL-6 is produced by monocytes infiltrating the myocardium and differentiating into inflammatory macrophages, contributing to tissue degradation and T cell activation.^4^ Previous studies have also demonstrated a ROS-inducing effect of IL-6 on cardiomyocytes and endothelial cells, comparable to our findings.^39,41^ Disturbances in calcium homeostasis through IL-6 have also been previously described in rat hearts.^40^ IL-6 inhibitors tocilizumab and sarilumab have been administered successfully to patients with severe COVID-19 to mitigate the excessive cytokine response, improving survival.^56^

Our study identified numerous proteins that had either not been previously correlated with severe iCMP or had only been minimally explored within this context. These included Collectin-12 (COLEC-12)—a scavenger receptor that plays a role in host defence. A pathomechanistic link to myocarditis, such as ROS induction, has not been identified in the literature to date. Similarly, the finding that COLEC-12 disrupts calcium homeostasis in cardiomyocytes represents a novel insight.

CRIM-1, a protein known for its association with arrhythmias,^38,43^ appears as a plausible contributor to the pathomechanism of severe heart failure through ROS induction and destabilization of calcium homeostasis.

Another protein that deserves mention is follistatin like 3 (FSTL3), an inhibitor of several members of the transforming growth factor β (TGF-β) superfamily, which was recently identified as a relevant prognostic marker for major adverse cardiovascular events in stable coronary artery disease. Higher levels of FSTL3 were associated with poor outcomes.^57^ Also in the current study, higher blood levels of FSTL3 were associated with severe iCMP.

Galectin 9 (LGALS9) is a protein that modulates cell–cell and cell–matrix interactions. It has been shown to promote innate immune cell activation by potentiating or synergizing toll-like receptor signalling.^58^ Also, LGALS9 has been associated with the severity of myocarditis in mice infected with Coxsackievirus B3 and to have an immunomodulatory effect when administered in experimental myocarditis.^59^

Our study included an analysis of real-world EMR data to examine whether blockade of the identified cytokines or their downstream signalling molecules through clinically available cytokine-inhibitors may have potential cardioprotective effects. Janus kinase inhibitors block downstream signalling of inflammatory pathways of IL-6 and other cytokines. Results from our current EMR data investigation demonstrating lower NT-proBNP and hs-TnT levels in patients receiving IL-6 or Janus kinase inhibitors are consistent with findings from previous studies in murine models suggesting that these drug classes may reduce cardiac inflammation and injury in both experimental and clinical settings.^42,52,60,61^ These data support the hypothesis that the relationship between identified cytokines and severe iCMP could involve causal mechanisms. These real-world data are subject to important limitations. In particular, indication bias and residual confounding from underlying rheumatologic disease, comorbidities, and concurrent therapies may have influenced the observed associations. Therefore, the apparent cardioprotective effects of IL-6 or JAK inhibition should be interpreted cautiously and require confirmation in *in vitro* and *in vivo* models and independent randomized patient cohorts with iCMP.

Our findings have potential implications for investigations of future treatment of patients with iCMP who clinically deteriorate despite currently available state-of-the art medical therapy. Cytokines may represent feasible therapeutic targets as prior research has shown. Blocking of cytokines, their receptors, or intracellular kinases downstream of cytokine signalling has been successfully applied.^4,16,19,20,21,22^ Also, removal through immunoadsorption or -filtration could represent another approach. Drug-repurposing of already existing drugs that target the cytokines of interest such as Ixekizumab and Secukinumab to neutralize IL17A may be tested in future experiments.

Translational perspective Cytokine profiling in a national clinical cohort of 425 patients with inflammatory cardiomyopathy identified multiple proteins associated with reduced LVEF. From these, three proteins (COLEC-12, CRIM-1, and IL-6) were selected for further testing based on biological plausibility, existing evidence, and their strong association with severe inflammatory cardiomyopathy. In cell culture experiments using induced pluripotent stem cell–derived cardiomyocytes and human aortic endothelial cells, these cytokines increased oxidative stress and disturbed calcium balance in cardiomyocytes, suggesting a possible role in cardiac injury. While inhibition of these cytokines may be technically achievable, our current findings are exploratory and will require confirmation through additional preclinical and clinical research.

## 5. Limitations

Our study highlighted several cytokines as potential novel therapeutic targets in iCMP, which could be relevant for patients whose clinical condition worsens as they do not respond to current optimal medical therapy. To clarify causal relationship and identify the most promising future targets among those cytokines we reported, further basic science experiments *in vitro* as well as in 3D- tissue-engineered cultures and animal models will be necessary. The use of iPSC-derived cardiomyocytes and human aortic endothelial cells represents some limitations, as relevant differences may occur depending on the specific differentiation protocol applied. In our study, we have used a well-established differentiation protocol as described by Dai *et al*.,^31,36^ Joseph C. Wu *et al*.,^62^ and Ebert *et al*.^33^ Differentiation was confirmed by immunostaining and RT-PCR for cardiac genes. Furthermore, studies of interindividual variation in cytokine receptor expression, potential side effects of cytokine inhibition, and long-term treatment effects should be investigated. Additional proteins identified in our study to have strong association with severe iCMP, including PLAUR, AGRN, and WNT9A, may constitute additional valuable targets for future mechanistic and translational research.

## 6. Conclusions

We tested the hypothesis that excess of specific cytokines in patients with severe iCMP may contribute to cardiac injury and result in reduced LVEF. Seventy-seven proteins were identified to be elevated in patients with severe iCMP. Furthermore, higher cytokine levels correlated with reduced LVEF. The data were statistically significant and replicated in an external national multicentre cohort (*n* = 435). Three top elevated proteins in iCMP, COLEC-12, CRIM-1, and IL-6 were chosen for further mechanistic testing in human iPSC-derived CMs and HAECs based on biological plausibility, support from existing literature, and their association with severe iCMP in our cohorts. Indeed, when those three cytokines were applied to iPSC-derived CMs and HAECs at concentrations observed in our cohort of severe iCMP, increased oxidative stress and calcium dysregulation was observed in particular in iPSC-derived CMs, consistent with a possible contribution to exacerbating cardiac dysfunction. Real-world data from an exploratory EMR analysis suggests that cytokine inhibitors already in clinical use for other inflammatory diseases, targeting the cytokines we identified or their downstream signalling pathways, may have a cardioprotective effect. These findings are consistent with our hypothesis, although further validation is needed.

Some of the cytokines identified in our study have previously been reported as potential therapeutic targets in inflammatory cardiomyopathy and heart failure in general, supporting the biological plausibility of our findings. In addition, we discovered several novel proteins of interest that may represent potential therapeutic targets via approaches such as binding and neutralizing agents, filtration, immunoadsorption, or repurposing of existing drugs. Such strategies could be relevant for individuals with limited response despite current state-of-the-art therapies.

## Supplementary Material

cvag099_Supplementary_Data

## Data Availability

An anonymized dataset can be made available upon request. Access is only granted to academic institutions and after signing a data sharing agreement. The code of the statistical evaluation for the statistics programme R is published on GitHub.
