# Supplementary material for: Cytokine-associated ROS and Ca2+ dysregulation in severe inflammatory cardiomyopathy
Source: Cardiovasc Res. 2026 Aug 30;122(14):1978–91. doi: 10.1093/cvr/cvag099 (PMC13653499; doi:10.1093/cvr/cvag099)
Supplement: Supplementary file 1 [file cvag099_supplementary_data.docx]

# **Supplement**

**Supplemental Table 1:** Deutsches Herzzentrum der Charité (DHZC) cohort (n=63): Top 10 cytokines associated with left ventricular ejection fraction (LVEF). Higher relative cytokine concentrations were associated with lower LVEF values. This inverse relationship with LVEF was significant for 5 cytokines (FDR < 0.05; FDR was calculated using the Benjamini-Hochberg procedure based on p-value from linear regression model). Estimate, the coefficient estimate from the linear regression model; r², coefficient of determination (r²) value for the model; P-value, raw P-value for the model; FDR, false discovery rate calculated using Benjamini-Hochberg correction.

| **Cytokine** | **Estimate** | **r2** | **P-value** | **FDR** |
| --- | --- | --- | --- | --- |
| VEGFD | -15,418 | 0,421638 | 5,47E-08 | 2,01E-05 |
| CLSTN2 | -12,3104 | 0,315359 | 1,01E-05 | 0,003696 |
| FSTL3 | -14,615 | 0,313893 | 1,08E-05 | 0,00394 |
| KRT19 | -8,64163 | 0,31284 | 1,13E-05 | 0,004121 |
| CRIM1 | -20,57 | 0,287229 | 3,54E-05 | 0,012901 |
| COLEC12 | -14,5942 | 0,244778 | 0,000219 | 0,079625 |
| CHRDL1 | -13,612 | 0,228297 | 0,000436 | 0,15771 |
| LAIR1 | -10,3162 | 0,222985 | 0,000542 | 0,195762 |
| PRSS8 | -12,4807 | 0,22271 | 0,000548 | 0,197437 |
| LY6D | -11,7192 | 0,217577 | 0,000677 | 0,243021 |

**Supplemental Table 2:** National cohort (n=425): Similar to the Deutsches Herzzentrum der Charité (DHZC) cohort, there was an inverse relationship between cytokine concentrations and left ventricular ejection fraction (LVEF) with high cytokine levels being associated with lower LVEF. Four out of five of the identified cytokines from the DHZC cohort were replicated in the national cohort. COLEC-12, ranked as the 6th most associated cytokine with LVEF in the DHZC cohort (P-value: 0.000219, FDR: 0.079625), emerged as the top-ranked cytokine in the national cohort (P-value: 4.46E-16, FDR: 1.64E-13). Overall, there were 77 cytokines significantly associated with LVEF (FDR < 0.05; FDR was calculated using the Benjamini-Hochberg procedure to p-values derived from the linear regression model). Estimate, the coefficient estimate from the linear regression model; r², coefficient of determination (r²) value for the model; P-value, raw P-value for the model; FDR, false discovery rate calculated using Benjamini-Hochberg correction.

| **Cytokine** | **Estimate** | **r2** | **P-value** | **FDR** |
| --- | --- | --- | --- | --- |
| COLEC12 | -12,1741 | 0,164675 | 4,46E-16 | 1,64E-13 |
| PLAUR | -11,1866 | 0,163439 | 6,12E-16 | 2,24E-13 |
| CCL3 | -7,14421 | 0,152285 | 1,04E-14 | 3,81E-12 |
| LILRB4 | -9,01034 | 0,151545 | 1,25E-14 | 4,58E-12 |
| CXCL17 | -5,54511 | 0,149364 | 2,18E-14 | 7,92E-12 |
| AGRN | -10,9161 | 0,148426 | 2,75E-14 | 1E-11 |
| WNT9A | -9,78385 | 0,141907 | 1,41E-13 | 5,11E-11 |
| CD4 | -11,4165 | 0,137435 | 4,31E-13 | 1,56E-10 |
| FABP1 | -4,20844 | 0,136129 | 5,96E-13 | 2,15E-10 |
| LAIR1 | -7,74702 | 0,133987 | 1,01E-12 | 3,64E-10 |
| LGALS9 | -8,96591 | 0,123841 | 1,24E-11 | 4,44E-09 |
| CRIM1 | -12,4333 | 0,122375 | 1,78E-11 | 6,35E-09 |
| TNFSF13 | -10,0253 | 0,121528 | 2,19E-11 | 7,79E-09 |
| FSTL3 | -7,4228 | 0,113642 | 1,5E-10 | 5,33E-08 |
| CCL7 | -4,43945 | 0,109818 | 3,8E-10 | 1,35E-07 |
| TGFA | -7,94094 | 0,109175 | 4,44E-10 | 1,57E-07 |
| CLSTN2 | -6,20831 | 0,107495 | 6,67E-10 | 2,35E-07 |
| HGF | -4,49271 | 0,104511 | 1,37E-09 | 4,82E-07 |
| ANGPTL4 | -6,68144 | 0,104232 | 1,47E-09 | 5,14E-07 |
| SPON1 | -7,40211 | 0,103125 | 1,92E-09 | 6,69E-07 |
| IL17D | -9,61421 | 0,101249 | 3,01E-09 | 1,05E-06 |
| LRRN1 | 7,205829 | 0,101132 | 3,1E-09 | 1,08E-06 |
| LY6D | -7,43169 | 0,098996 | 5,18E-09 | 1,79E-06 |
| PON3 | 11,39412 | 0,095766 | 1,13E-08 | 3,88E-06 |
| HLA-E | -11,4467 | 0,095722 | 1,14E-08 | 3,91E-06 |
| SPINK4 | -4,40881 | 0,095501 | 1,2E-08 | 4,11E-06 |
| LGALS4 | -5,27358 | 0,09436 | 1,58E-08 | 5,39E-06 |
| IL6 | -2,99671 | 0,093813 | 1,8E-08 | 6,13E-06 |
| NPPC | -4,98094 | 0,093211 | 2,08E-08 | 7,06E-06 |
| CHRDL1 | -7,91593 | 0,092942 | 2,21E-08 | 7,51E-06 |
| TREM2 | -4,77254 | 0,091431 | 3,18E-08 | 1,07E-05 |
| CXCL10 | -4,17866 | 0,088631 | 6,21E-08 | 2,09E-05 |
| EPO | -3,74666 | 0,087718 | 7,72E-08 | 2,59E-05 |
| CXCL8 | -4,01822 | 0,087691 | 7,77E-08 | 2,6E-05 |
| MATN2 | -9,39644 | 0,086945 | 9,28E-08 | 3,1E-05 |
| CXCL14 | -4,49222 | 0,08555 | 1,29E-07 | 4,31E-05 |
| GAL | 4,65288 | 0,082948 | 2,4E-07 | 7,98E-05 |
| TFF2 | -4,06551 | 0,081606 | 3,31E-07 | 0,000109 |
| PREB | -10,3113 | 0,081157 | 3,68E-07 | 0,000121 |
| SULT2A1 | -4,04533 | 0,078762 | 6,5E-07 | 0,000214 |
| ENPP7 | -3,32201 | 0,077855 | 8,06E-07 | 0,000264 |
| IL15 | -6,78804 | 0,077687 | 8,39E-07 | 0,000274 |
| DNER | 9,589636 | 0,077289 | 9,22E-07 | 0,0003 |
| CXCL9 | -3,22193 | 0,074958 | 1,6E-06 | 0,000521 |
| IL4R | -6,07854 | 0,074123 | 1,95E-06 | 0,000633 |
| REG4 | -4,97668 | 0,073114 | 2,48E-06 | 0,000801 |
| BTN3A2 | -6,09672 | 0,071531 | 3,61E-06 | 0,001162 |
| FST | -5,18705 | 0,071502 | 3,63E-06 | 0,001166 |
| EGLN1 | -3,95482 | 0,07108 | 4,01E-06 | 0,001284 |
| TNF | -5,94492 | 0,070676 | 4,42E-06 | 0,001409 |
| IL1RN | -3,4451 | 0,070089 | 5,08E-06 | 0,001614 |
| NTF3 | -5,07255 | 0,069915 | 5,29E-06 | 0,001677 |
| TNFRSF13B | -6,8624 | 0,069553 | 5,76E-06 | 0,001821 |
| CXADR | -4,82297 | 0,068877 | 6,76E-06 | 0,00213 |
| CD276 | -5,47858 | 0,068871 | 6,77E-06 | 0,00213 |
| TNFRSF11A | -4,2661 | 0,06851 | 7,38E-06 | 0,002308 |
| SMOC2 | -5,99596 | 0,068308 | 7,73E-06 | 0,002413 |
| CCL25 | -4,11859 | 0,067723 | 8,88E-06 | 0,002763 |
| NFASC | -7,98799 | 0,067721 | 8,89E-06 | 0,002763 |
| CCL28 | -3,05271 | 0,067265 | 9,9E-06 | 0,003059 |
| SIGLEC10 | -5,64472 | 0,066844 | 1,09E-05 | 0,003368 |
| PRSS8 | -5,97756 | 0,066519 | 1,18E-05 | 0,003626 |
| LAMA4 | -6,9675 | 0,066442 | 1,2E-05 | 0,00368 |
| PRELP | -8,7097 | 0,065305 | 1,57E-05 | 0,004801 |
| ENPP5 | 5,817113 | 0,063325 | 2,51E-05 | 0,007643 |
| CCL21 | -4,3005 | 0,063229 | 2,57E-05 | 0,007793 |
| TNFRSF4 | -4,64774 | 0,061676 | 3,71E-05 | 0,011215 |
| HLA-DRA | -5,76163 | 0,061424 | 3,94E-05 | 0,011865 |
| CCL23 | -5,004 | 0,061069 | 4,29E-05 | 0,012863 |
| FASLG | 4,476295 | 0,060662 | 4,72E-05 | 0,014115 |
| TPP1 | -5,9269 | 0,059997 | 5,53E-05 | 0,016466 |
| CCL11 | -4,03204 | 0,059247 | 6,6E-05 | 0,019599 |
| CCL13 | -2,60901 | 0,057756 | 9,39E-05 | 0,0278 |
| KRT19 | -3,53618 | 0,057209 | 0,000107 | 0,031541 |
| LIFR | -6,6545 | 0,05658 | 0,000124 | 0,036485 |
| ITM2A | -4,51045 | 0,056553 | 0,000125 | 0,036589 |
| CKAP4 | -5,15816 | 0,056407 | 0,000129 | 0,037749 |

**Supplemental Table 3:** Replication in the national cohort confirms that higher relative cytokine concentrations of the top 10 cytokines from the derivation cohort are associated with lower LVEF in patients with inflammatory cardiomyopathy. Estimate, the coefficient estimate from the linear regression model; r², coefficient of determination (r²) value for the model; P-value, raw P-value for the model; FDR, false discovery rate calculated using Benjamini-Hochberg correction.

| **Cytokine** | **Estimate** | **r2** | **P-value** | **FDR** |
| --- | --- | --- | --- | --- |
| COLEC12 | -12.17 | 0.1647 | 4.5e-16 | 1.6e-13 |
| LAIR1 | -7.747 | 0.134 | 1.0e-12 | 3.6e-10 |
| CRIM1 | -12.43 | 0.1224 | 1.8e-11 | 6.3e-09 |
| FSTL3 | -7.423 | 0.1136 | 1.5e-10 | 5.3e-08 |
| CLSTN2 | -6.208 | 0.1075 | 6.7e-10 | 2.3e-07 |
| LY6D | -7.432 | 0.099 | 5.2e-09 | 1.8e-06 |
| CHRDL1 | -7.916 | 0.09294 | 2.2e-08 | 7.5e-06 |
| PRSS8 | -5.978 | 0.06652 | 1.2e-05 | 0.0036 |
| KRT19 | -3.536 | 0.05721 | 0.00011 | 0.0315 |
| VEGFD | -3.324 | 0.04488 | 0.00202 | 0.5383 |

**Supplement table 4: Results of Quantitative H2DCFDA Assay in Cardiomyocytes**

* indicates a significant increase in ROS production measured by H2DCFDA assay with a

P-value below 0.05, ** a P-value below 0.01, or *** a P-value below 0.001 in comparison to the control samples.

| Timepoint | Cytokine | P-value | Significance |
| --- | --- | --- | --- |
| t24h | IL-6 | 0.000796 | *** |
| t24h | CRIM-1 | 0.016879 | * |
| t24h | COLEC-12 | 0.0006 | *** |
| t48h | IL-6 | 0.006905 | ** |
| t48h | CRIM-1 | 0.002131 | ** |
| t48h | COLEC-12 | 0.00322 | ** |

**Supplement table 5: Results of Quantitative H2DCFDA Assay in HAEC**

* indicates a significant increase in ROS production with a P-value below 0.05, ** a P-value below 0.01, and *** a P-value below 0.001 in comparison to the control samples.

| Timepoint | Cytokine | P-value | Significance |
| --- | --- | --- | --- |
| t24h | COLEC-12 | 1.16E-05 | *** |
| t24h | CRIM-1 | 0.004929 | ** |
| t24h | IL-6 | 0.001546 | ** |
| t48h | IL-6 | 0.002764 | ** |
| t48h | CRIM-1 | 0.03493 | * |
| t48h | COLEC-12 | 0.020444 | * |

**Supplemental Figure 1:** A-B, Human iPSC-CMs were subjected to immunostaining and confocal imaging for cardiomyocyte markers. A, cardiac troponin T (TNNT2), myosin binding protein C (MYBPC3); B, TNNT2, sarcomeric α-actinin (SAA). Representative 63x confocal images are shown.

**Supplemental Figure 2:** Relative messenger ribonucleic acid (mRNA) expression of cardiomyocyte marker genes in induced pluripotent stem cell–derived cardiomyocytes (iPSC-CM) and human aortic endothelial cells (HAEC) assessed by reverse transcriptase real-time polymerase chain reaction (RT-PCR). Gene targets include cardiac troponin T type 2 (TNNT2), cardiac troponin I type 3 (TNNI3), myosin heavy chain 7 (MYH7), actin alpha cardiac muscle 1 (ACTC1), and myosin heavy chain 2 (MYH2). Relative expression was normalized to the housekeeping gene 18S ribosomal ribonucleic acid (18S rRNA). Data are shown as individual measurements with boxplots indicating median and interquartile range; whiskers represent the range within 1.5 × interquartile range. Relative expression values are presented as 2^ΔΔCt^. RT-PCR analysis was conducted in a single run, with technical duplicates for HAEC samples and technical quadruplicates for iPSC-CMs.

**Supplemental Figure 3:** NPX values of all protein assays in the first batch (X axis, derivation and generalizability cohorts) and second batch (Y axis, validation cohort) data sets for the 16 bridging samples. Each dot represents one protein assay in one sample. Left panel, raw data. Right panel, data normalized using the olink_normalization function from the OlinkAnalyze R package. In total, there were bridge 16 samples, 368 assays and 5888 total measurements. Blue line shows a fitted LOESS model. For this analysis each sample was analyzed in duplicate to ensure technical consistency.

**Supplemental Figure 4:** Comparison of 10 randomly chosen protein assays across the different data sets and different normalizations for the 16 bridging samples. Top: Raw data. Bottom: Normalized data. In total, 16 unique samples and 640 measurements are shown. For this analysis each sample was analyzed in duplicate to ensure technical consistency.

**Supplemental Figure 5:** Principal component analysis of the 488 measurements before normalization (left panel) and the same measurements after normalization (right panel). Each dot represents one sample. The first two principal components are shown in the top row, and the third and fourth principal components are shown in the bottom row. The color of the dots indicates the batch of the sample.

Supplemental Figure **6**: Scree plot of the principal component analysis of the 488 measurements before normalization (red) and the same measurements after normalization (blue). The x-axis depicts the principal components and the y-axis depicts the proportion of variance explained by each principal component.

Supplemental Figure 7: Schematic representation of the experimental setup for cell culture assays, illustrating the measurement of ROS production and calcium influx and efflux in induced human pluripotent stem cell-derived cardiomyocytes (iPSC-derived CMs) and human aortic endothelial cells (HAECs).

Supplemental Figure 8: Oxidative Stress During Cytokine Exposure in iPSC-Derived Cardiomyocytes**:** The bar chart depicts reactive oxygen species (ROS) production in induced human pluripotent stem cell-derived cardiomyocytes (iPSC-Derived CMs) after 24h and 48h exposure to individual cytokines: COLEC-12, CRIM-1, or IL-6. The positive control, cells treated with hydrogen peroxide, consistently exceeded control levels in all experiments. Data are presented as the mean with a 95% confidence interval. All results were statistically significant when compared to controls (see supplement table 4) FC: Fold change. P<0,001***; P <0,01**; P<0,05*. A t-test was used to compare the individual cytokine with control. Each experiment was independently repeated twice using the same cell line. Within each experiment, all conditions and controls were measured in technical quadruplicates.

Supplemental Figure 9: Oxidative Stress During Cytokine Exposure in Human Aortic Endothelial Cells: The bar chart illustrates ROS production in human aortic endothelial cells (HAEC) after 24h and 48h exposure to individual cytokines: COLEC-12, CRIM-1, or IL-6. The positive control, cells treated with hydrogen peroxide, consistently exceeded control levels in all experiments. Data are presented as the mean with a 95% confidence interval. All results were statistically significant compared to controls (see supplement table 5). FC: Fold change. P<0,001***; P <0,01**; P<0,05*. A t-test was used to compare the individual cytokine with control. Each experiment was independently repeated twice using the same cell line. Within each experiment, all conditions and controls were measured in technical quadruplicates.
